# Reproductive trade-offs maintain bract color polymorphism in Scarlet Indian paintbrush (*Castilleja coccinea*)

**DOI:** 10.1371/journal.pone.0209176

**Published:** 2019-01-02

**Authors:** Eun Sun Kim, David N. Zaya, Jeremie B. Fant, Mary V. Ashley

**Affiliations:** 1 Department of Biological Sciences, University of Illinois at Chicago, Chicago, Illinois, United States of America; 2 Illinois Natural History Survey, Champaign, Illinois, United States of America; 3 Institute for Plant Conservation, Chicago Botanic Garden, Glencoe, Illinois, United States of America; 4 Plant Biology and Conservation, Northwestern University, Evanston, Illinois, United States of America; University of North Carolina at Greensboro, UNITED STATES

## Abstract

Populations of scarlet Indian paintbrush (*Castilleja coccinea*) in the Midwestern United States exhibit a bract color polymorphism, with each population having predominantly yellow or scarlet bracts. We investigated a possible mechanism for this maintenance of bract color polymorphism in *C*. *coccinea* by conducting hand-pollination experiments in two nearby populations, one predominantly yellow and one predominantly scarlet. The hand-pollination treatments were either self-pollination or cross pollination using pollen from within and between populations. Both color morphs were used as pollen donors for the within and between crosses. We found that both color morphs of *C*. *coccinea* were self-compatible. When the scarlet morph was the maternal plant it had higher seed set. When pollinators were excluded, the yellow morph outperformed the scarlet morph in fruit set and seed set. The apparent trade-offs between a higher reproductive output in the scarlet morph and a reproductive assurance advantage in the yellow morph may explain the maintenance of the polymorphism in *C*. *coccinea*. While many previous studies have provided evidence for pollinator preference playing a role in floral color polymorphism, the results of the current study indicate that reproductive assurance, which would be important for fluctuations in pollinator abundance or colonizing new areas, may act as a selective agent to maintain such polymorphisms.

## Introduction

Polymorphisms for floral traits occur in many angiosperm species, and the underlying evolutionary forces maintaining these polymorphisms have long been the subject of interest and debate among evolutionary biologists. Floral traits reported to vary intraspecifically include corolla length and corolla flare [1], calyx length [2], flower size and style length [3], and floral color [4–6]. Among these traits, floral color polymorphisms are the most visually striking and thus have drawn many researchers to investigate the cause and maintenance of intraspecific variation [7–10]. Floral color polymorphisms vary both within [5,11–14] and between populations [4,15–17] and a variety of selective agents have been implicated in their maintenance.

Numerous studies have demonstrated that pollinators are often the primary selective agent maintaining floral color polymorphisms both within and between populations [11,18–23]. Pollinator preference and constancy may result in assortative mating, limiting gene flow between the morphs within a population [24,25]. For *Ipomoea purpurea*, pollinator constancy by bumble bees resulted in assortative mating within a population [26], while in *Clarkia xantiana*, floral color polymorphism is maintained via a combination of positive frequency-dependent pollinator preference by one bee species and negative frequency-dependent pollinator preferences by two other bee species [13]. In *Mimulus aurantiacus*, where red and yellow ecotypes inhabit different habitats, hummingbirds and hawkmoths show strong preference for red and yellow morphs, respectively, hence both pollinator preferences and ecogeographic isolation has led to assortative mating, thereby maintaining the flower color polymorphism between populations [27].

Selection by non-pollinator agents can also lead to floral color polymorphism [28,29]. Differences in seed set, seed weight, and seed predation under different environmental conditions have been documented between color morphs [4,30–32]. Anthocyanins, a primary floral pigment [33], are related to tolerance against abiotic stresses such as UV-B radiation [34], heat [35], and drought [36], as well as non-pollinator biotic pressures such as herbivore defense [6,37]. Such pleiotropic effects will interact with the pollinator community to either maintain or enforce floral color polymorphism [38].

In theory, floral color polymorphisms associated with differences in breeding system could also be maintained by selection. For example, autogamous selfing (within the same flower) provides reproductive assurance when vector-mediated cross pollination is insufficient, but the advantage is offset by pollen and/or seed discounting [39,40]. Color morphs associated with higher rates of selfing may therefore have a selective advantage when pollinators are limited but not when they are abundant. Numerous studies have demonstrated that intraspecific variation in other floral traits, such as herkogamy and protandry [41], flower size [42,43] and even scent [44] can influence reproductive assurance within and between populations. We are aware of only one report of differences in selfing rates associated with variation in flower color. In *Ipomoea purpurea*, when the relative frequency of the white morph is low compared to the darkly and lightly pigmented morphs, the white morph had higher selfing rates [26,45]. When morphs were at more similar frequencies, all three morphs had similar selfing rates, so the white morph seems to be maintained by negative frequency dependent selection on reproductive assurance [45–47].

Scarlet Indian paintbrush, *Castilleja coccinea* L. Sprengel (Orobanchaceae), is a hemiparasitic forb native to the Eastern United States. Showy bracts surround small, greenish flowers. Flowers are perfect with the style slightly exserted [48]. Individuals are annual or biennial and may produce multiple stems and inflorescences. A successful fertilization results in a capsule that contains an average of 150 seed. The bracts surrounding flowers display yellow or scarlet (orange-red) colors. Despite the common name, the yellow morph dominates some populations in the Midwestern United States. Populations in this region are predominantly one color or the other, with over 90% of the individuals typically having either yellow or scarlet bract colors [49]. Although the basis of bract color has not been studied, seeds collected from natural populations and sown in a common garden grew into plants exhibiting maternal bract colors, suggesting that bract color is a heritable trait [49]. Further, we have hand pollination data that indicates bract color shows simple Mendelian inheritance, with yellow dominant over scarlet (in prep). *Castilleja coccinea* has been reported to attract ruby-throated hummingbirds, *Archilochus colubris* [50–54] and insect pollinators such as bees and butterflies. It is tempting to hypothesize that pollinator preference might cause positive assortative mating and thus maintains the bract color polymorphism in *C*. *coccinea*, but there are no published studies demonstrating different rates of pollinator visitations and effectiveness to color morphs in this species.

We investigated the possible role of the breeding system in the maintenance of flower color polymorphism in *C*. *coccinea*. We used hand-pollination experiments at a site in northeastern Illinois (Illinois Beach State Park) where a yellow population and a scarlet population are found approximately 500 m apart. The color morphs grow on the same sandy dune-swale complex under similar abiotic conditions, and likely share a pollinator community. Our overall goal was to characterize the breeding system of the species, and to identify differences between color morphs, if they occurred. We used pollinator exclusion and hand-pollination experiments to compare the color morphs with regard to 1) self-compatibility, 2) response to pollinator exclusion, 3) cross-compatibility between the color morphs, and 4) relative female fertility and male fitness.

## Materials and methods

### Study populations

A hand-pollination experiment was conducted at Illinois Beach State Park from May 29^th^ to July 6^th^ in 2013. Two populations in Illinois Beach State Park, separated by an oak savannah and approximately 500 m apart, differ in bract color. Population 1 (hereafter, the yellow population) is predominantly yellow (87% yellow) whereas population 2 (hereafter, the scarlet population) is predominantly scarlet (99.6% scarlet) [49]. A limited pollinator observation study was conducted in both populations to determine the presence or absence of floral visitors to *C*. *coccinea*. A total of 24 observation sessions, each lasting 15 minutes, were conducted in the yellow population, and 17 observation sessions were conducted in the scarlet population from morning to late afternoon.

### Hand-pollinations

**Fig 1:**
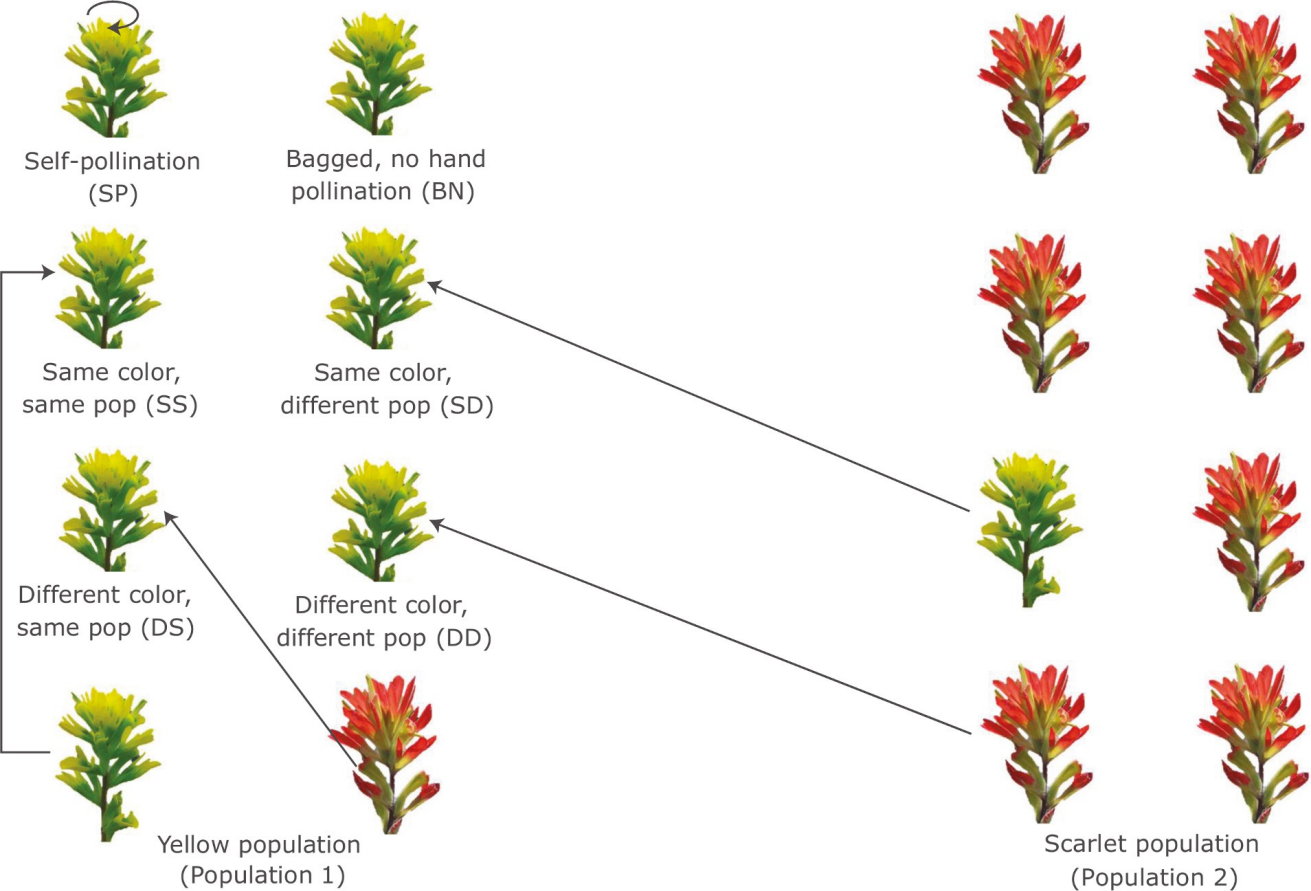
Illustration of six hand-pollination treatments for the yellow morph. All pollen recipients were bagged. The arrows move away from pollen donors and point to pollen recipients. The scarlet morph also received the corresponding six treatments but are not illustrated in this figure. There were three replicates for each treatment.

We conducted hand-pollinations to study the breeding system of *C*. *coccinea* and to compare female fertility under different treatments. To exclude animal pollinators, we used nylon mesh bags (17.8 cm by 11.4 cm) to cover entire inflorescences for all six treatments. The pollen donors varied by bract color and population. There were six pollination treatments: 1) bagged, no hand pollination (BN); 2) self-pollination (SP); 3) same color, same population (SS); 4) same color, different populations (SD); 5) different color, same population (DS); and 6) different color, different populations (DD) (Fig 1). For self-pollination, the pollen was transferred to the stigma of the same flower (autogamous selfing). For all “same population” treatments, pollen donors were chosen at least 5 m apart from the pollen recipients to decrease chances of biparental inbreeding. Eighteen randomly selected individuals from each population were chosen as pollen recipients, providing three replicates for all six treatments. All 18 individuals were defined by the predominant color of the population, hence the “yellow morph” refers to a pollen recipient with yellow bract color from the yellow population and the “scarlet morph” refers to a pollen recipient with scarlet bract color from the scarlet population. For the treatment assigned “same color, between populations”, the pollen donor was the same color as the predominate color of the recipient population but from the other population. Toothpicks and small plastic containers were used to remove and transfer pollen grains between individuals and populations. New pollen grains were collected for each day’s hand-pollination. Leftover pollen grains were discarded. In addition to the experimental treatments, three open control plants were followed in each population.

Flowers that were open prior to bagging were counted and marked by threads. Hand-pollinated flowers were marked with a black permanent marker. On each visit, the number of fertilized flowers was recorded for each individual. For the open control, the number of fertilized flowers were divided by the total number of flowers to estimate the fruit set. When individuals receiving treatments were either removed or had a broken stem, they were replaced with other individuals to keep sample size consistent. A total of 35 individuals were used for the fruit set analyses. One yellow morph that received the SS treatment had a broken stem, but it was too late in the blooming season to replace it. After collecting the fruit set data, two additional individuals were found with broken stems, one yellow morph that received the DD treatment and one yellow morph that received the SD treatment. Excluding these two, a total of 33 individuals were used for the seed set analyses. To calculate seed set we collected mature capsules and counted the number of seeds for each capsule using a Contador seed counter (Pfeuffer GmbH, Kitzingen, Germany). After the seed count, the seeds were returned to the site.

### Data analysis

Two variables were used as metrics of reproductive success, fruit set (the proportion of flowers that developed into fruits) and seed set (the number of seeds per fruit). Seed set excluded flowers that did not develop into fruits. We used linear mixed-effects models (LMM) for analyses involving seed set and generalized linear mixed-effects models (GLMM) for analyses involving fruit set. For GLMM relating to fruit set, fertilization of individual flowers was modeled as a binary (Bernoulli) response variable. Mixed-effects modeling was implemented with the *lme4* package for R version 3.5.0 [55,56]. In all mixed-effects models, we used the individual plant as the random effect (random intercept), and maternal color as one of the fixed effects.

To address our questions regarding self-compatibility, we compared the reproductive success of plants that were self-pollinated to those that were cross-pollinated with plants of the same color and the same population (i.e., the SP and SS treatments, respectively). Self-compatibility indices were calculated for each population using the average seed set where the average seed set from SP was divided by the average seed set from SS [57]. To investigate the effects of pollinator exclusion, we compared the reproductive success of plants that were bagged and not hand-pollinated to those that were self-pollinated (i.e., the BN and SP treatments). For each model we included the interaction of treatment and bract color. Thus, the maximal model had two fixed effects (maternal color, pollination treatment) with two levels each, an interaction of the fixed effects, and a random effect of the individual.

Additionally, we compared the open control treatment (unmanipulated, unbagged plants) in each population. This analysis only included maternal color as a fixed effect (same as the source population).

We investigated cross-compatibility between color morphs with regard to the relative fitness of the sexes using a larger model, that added 1) whether pollen came from the same or different population, (i.e., the “population” fixed effect), and 2) whether pollen came from individuals of the same bract color, (i.e., the “color” fixed effect), in addition to maternal color. Both of these had two levels (same or different). These two fixed effects combined to describe four of the pollination treatments described earlier (SS, SD, DS, and DD). The interactions of maternal color with each of the other two fixed effects were also included in model testing.

For each test, we compared candidate models with every combination of fixed effects using the Akaike information criterion corrected for small sample size (AICc). Akaike weights were calculated and the best model, assuming one correct model exists in the candidate set, was determined by the maximum Akaike weight value. Estimates of mean values and 95% confidence intervals for a given factor in our mixed-effects models were presented as estimated marginal means calculated using the *lsmeans* package [58].

We determined the relative influence of fixed effects and random effects by comparing the marginal and conditional R^2^, following Nakagawa and Schielzeth [59]. Calculation of the coefficients of determination was completed with the *MuMIn* package [60].

## Results

Our limited pollinator observations confirmed the presence of insect floral visitors. In the yellow population, we observed black swallowtails (*Papilio polyxeneson*) and bumble bees (*Bombus* sp.) on the yellow morph. In the scarlet population, we observed only sweat bees on the scarlet morph. Bumble bees and butterflies were present in the scarlet population, but they did not visit scarlet individuals during our observation sessions. We did not observe ruby-throated hummingbirds at our study sites.

### Self-compatibility

**Fig 2:**
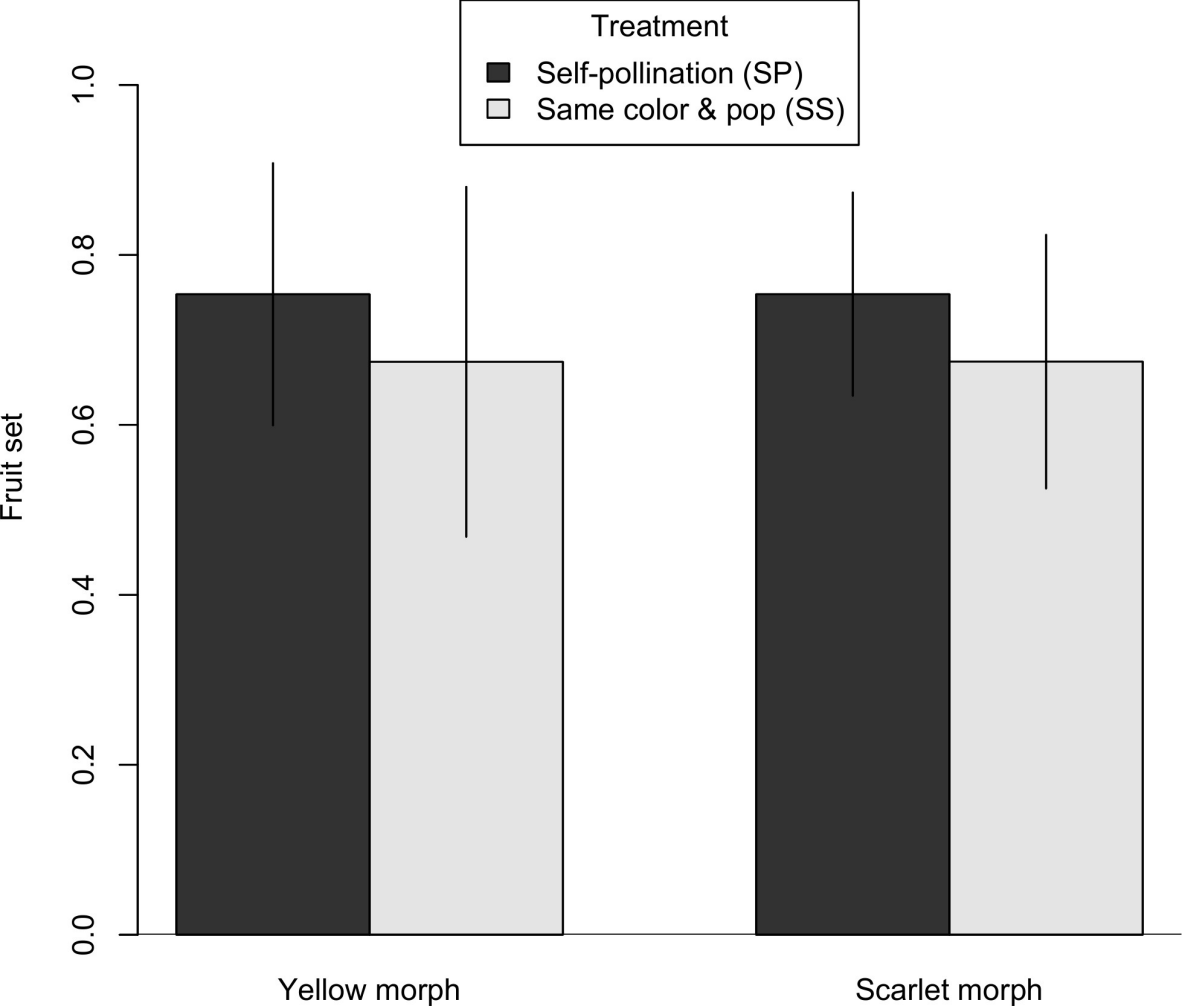
Fruit set comparison between self-pollination and outcrossing. For each maternal color, we compared fruit set in two treatments, SP and SS. Bar heights represent the estimated marginal means from the GLMM and error bars represent the 95% confidence interval.

Both color morphs were self-compatible with no evidence of self-sterility. The average seed set for SP was 110.43 and 144.43, for the yellow morph and the scarlet morph, respectively. The average seed set for SS was 121.89 and 162.40, for the yellow morph and the scarlet morph, respectively. The self-compatibility indices were 0.91 for the yellow and 0.89 for the scarlet. The self-pollination (SP) treatment had similar fruit set to the same color, same population (SS) treatment (Fig 2, S1A Table). The average fruit set was 60% in all treatment-color combinations and 70% for the self-pollination treatment (Fig 2). In our statistical models for fruit set (Table 1), the null model had the greatest support (Akaike weight = 0.447; Table 1). There was little support for a difference between treatments (ΔAICc = 1.31, Akaike weight = 0.232), or color morphs (ΔAICc = 2.11, Akaike weight = 0.155).

**Table 1:** Summary of model selection results for the relationship between fruit set and self-compatibility.

Model	df	AICc	ΔAICc	Weight
Null	2	131.7	0	0.447
Treatment	3	133	1.31	0.232
Maternal color	3	133.8	2.11	0.155
Maternal color + Treatment + Maternal color:Treatment	5	135	3.27	0.087
Maternal color + Treatment	4	135.2	3.47	0.079

Fruit set was predicted by two fixed effects and their interaction. This set of models includes the SP and SS treatments.

**Fig 3:**
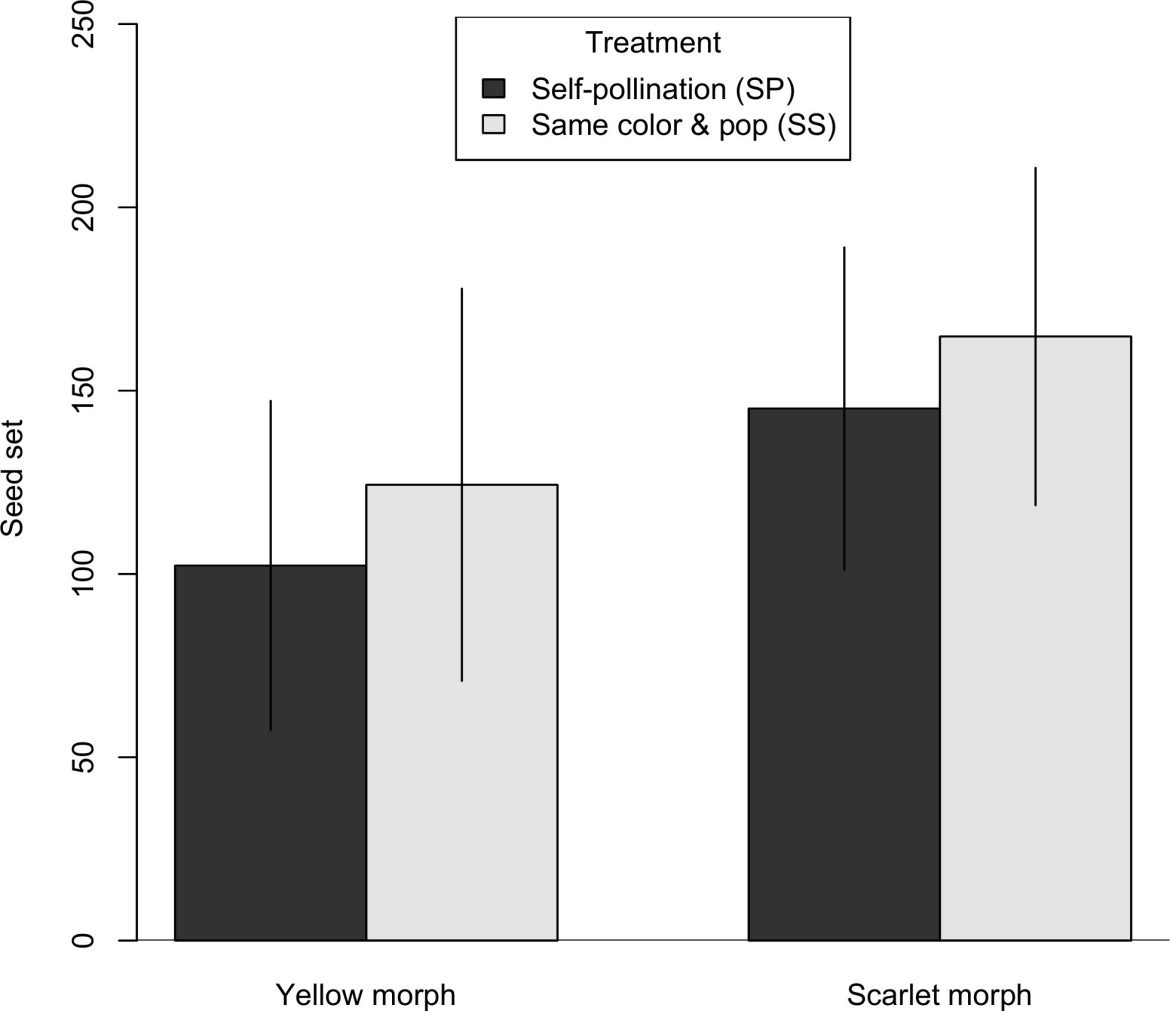
Seed set comparison between self-pollination and outcrossing. For each maternal color, we compared seed set in two treatments, SP and SS. Bar heights represent the estimated marginal means from the LMM and error bars represent the 95% confidence interval.

Seed set was about 18% lower in the self-pollination treatment (SP) compared to the same color, same population (SS) treatment (Fig 3, S1B Table), but there was little statistical support for including treatment in the best model (Table 2; for the best model with treatment, ΔAICc = 1.51, Akaike weight = 0.198). There was a more pronounced difference between color morphs, with scarlet individuals having 33% to 42% greater seed set for the cross-pollination (SS) and self-pollination (SP) treatments, respectively. The best model did include maternal color as a fixed effect (Akaike weight = 0.423), while the null model excluding maternal color and treatment was the next best model (ΔAICc = 1.37, Akaike weight = 0.213).

**Table 2:** Summary of model selection results for the relationship between seed set and self-compatibility.

Model	df	AICc	ΔAICc	Weight
Maternal color	4	540.8	0	0.423
Null	3	542.2	1.37	0.213
Maternal color + Treatment	5	542.4	1.51	0.198
Treatment	4	543.5	2.67	0.111
Maternal color + Treatment + Maternal color:Treatment	6	545	4.11	0.054

Seed set was predicted by two fixed effects and their interaction. This set of models includes the SP and SS treatments.

Among open control plants, one yellow plant had a broken stem and one scarlet plant could not be located after collecting the fruit set data (S1G and S1H Table). For the remaining plants, fruit set in the open control was not different between the two populations, as the null model was preferred (ΔAICc = 2.08, weight = 0.739). Fruit set for open control plants was high in both populations: 80.6% in the yellow population (95% confidence interval, 66.7%-94.6%) and 82.8% in the scarlet population (73.0%-92.5%). When testing seed set, model selection showed a slight preference for the model that included population over the null model (ΔAICc = 0.45, weight = 0.556). Seed set in the scarlet population was estimated as 188.8 seeds per fruit (95% C.I., 117.3–260.4), much greater than the 96.3 seeds per fruit (27.40086–165.2658) estimated for the yellow population.

### Pollinator exclusion

**Fig 4:**
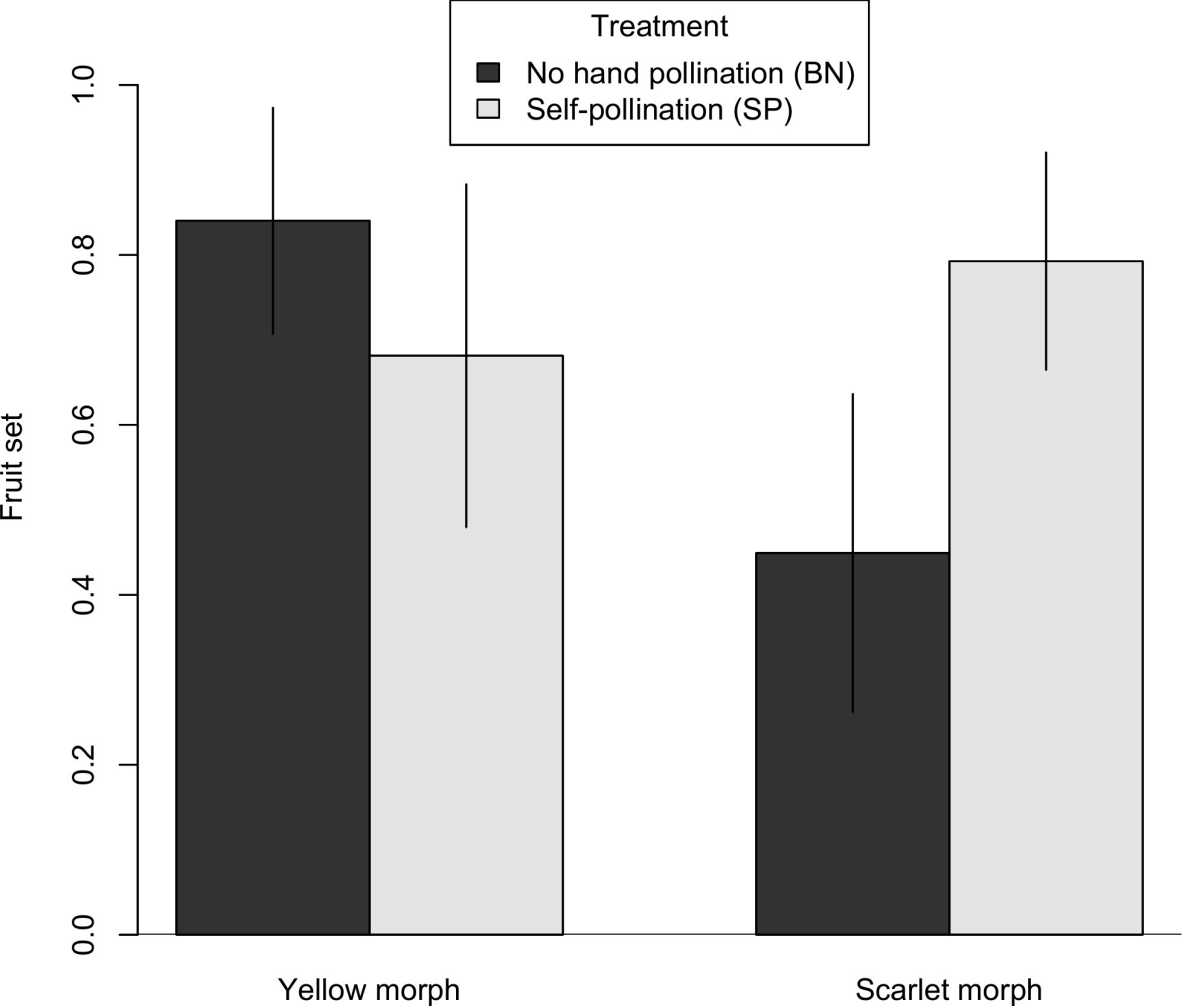
Fruit set comparison between bagged, no hand pollination and self-pollination. For each maternal color, we compared fruit set in two treatments, BN and SP. Bar heights represent the estimated marginal means from the GLMM and error bars represent the 95% confidence interval.

**Fig 5:**
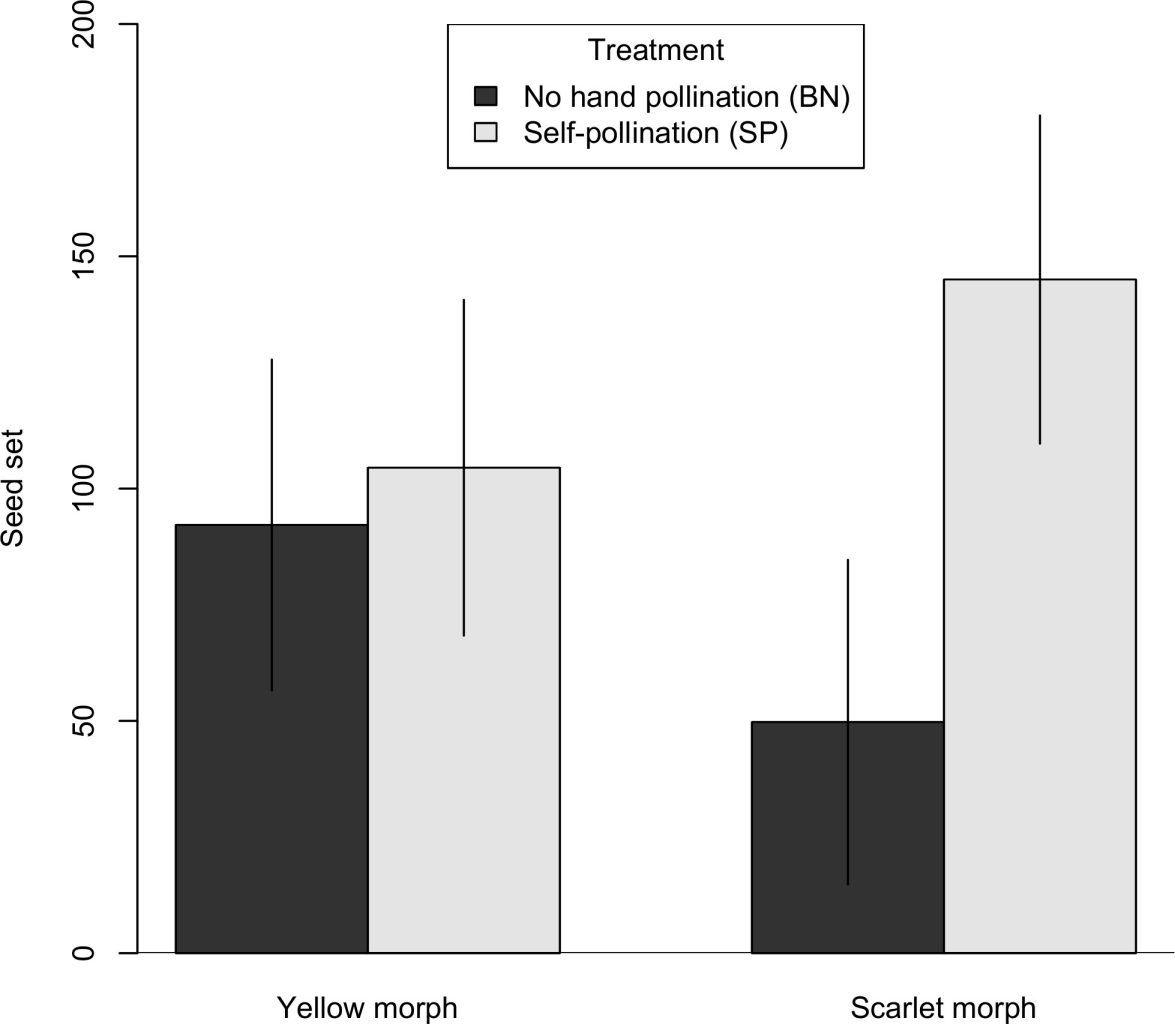
Seed set comparison between bagged, no hand pollination and self-pollination. For each maternal color, we compared seed set in two treatments, BN and SP. Bar heights represent the estimated marginal means from the LMM and error bars represent the 95% confidence interval.

Both morphs were capable of self-fertilization; both the bagged, no hand pollination treatments (BN) and the self-pollination (SP) treatments yielded fruits and seeds. Fruit and seed production in the BN treatment indicates either autonomous self-pollination, a bag effect (accidental pollination when bags were placed or removed), or pollination by “squatters” [57] (small, long-staying insects such as aphids and thrips already present when bags were placed). Interestingly, the color morphs differed markedly in comparisons of the BN and SP treatments. For the scarlet morph, the BN plants had a 43% reduction in fruit set (Fig 4, S1C Table) and a 66% decline in seed set compared to the SP plants (Fig 5, S1D Table). For the yellow morph, the BN plants had no reduction in fruit set (Fig 4), and only a 12% reduction in seed set relative to the SP plants (Fig 5). Thus, pollinator exclusion had a greater effect on the scarlet morph with respect to both fruit set and seed set (Figs 4 and 5). Statistical modeling provides the most support for difference between morphs with respect to reliance on a pollen vector. For both fruit set (Table 3) and seed set (Table 4), the best candidate model included the interaction between treatment and maternal color (fruit set, Akaike weight = 0.598; seed set, Akaike weight = 0.483).

**Table 3:** Summary of model selection results for the relationship between fruit set and pollinator exclusion.

Model	df	AICc	ΔAICc	Weight
Maternal color + Treatment + Maternal color:Treatment	5	155	0	0.598
Null	2	157.7	2.7	0.155
Maternal color	3	158.4	3.41	0.109
Treatment	3	159	4.08	0.078
Maternal color + treatment	4	159.5	4.58	0.061

Fruit set was predicted by two fixed effects and their interaction. This set of models includes the BN and SP treatments.

**Table 4:** Summary of model selection results for the relationship between seed set and pollinator exclusion.

Model	df	AICc	ΔAICc	Weight
Maternal color + Treatment + Maternal color: Treatment	6	619.4	0	0.483
Treatment	4	620.2	0.85	0.316
Maternal color + Treatment	5	622.6	3.25	0.095
Null	3	622.9	3.56	0.081
Maternal color	4	625.2	5.87	0.026

Seed set was predicted by two fixed effects and their interaction. This set of models includes the BN and SP treatments.

### Cross-compatibility and relative fitness

**Fig 6:**
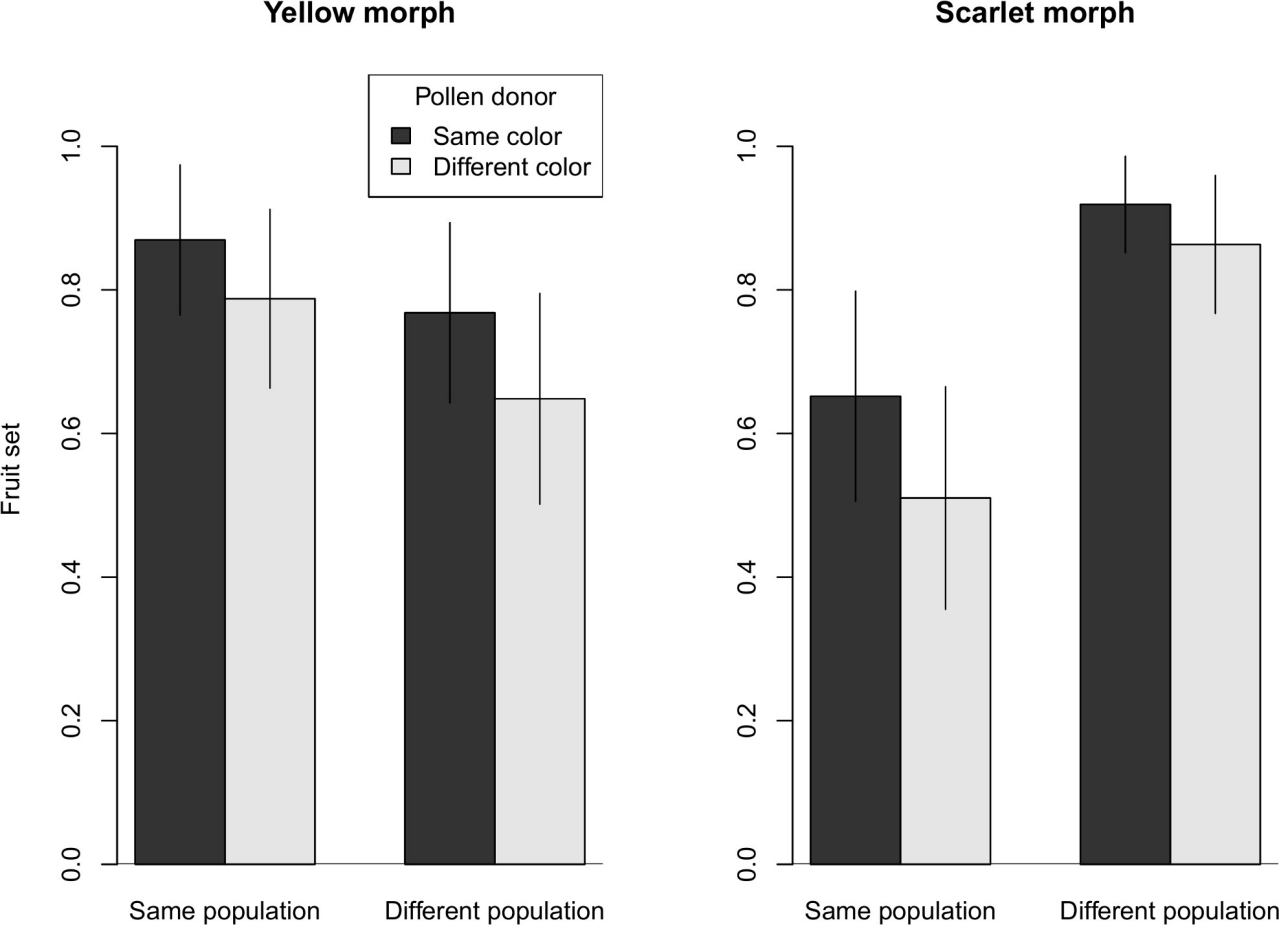
Fruit set comparison in inter-population and inter-morph crosses. For each maternal color, we compared fruit set in four treatments (SS, SD, DS, and DD) that combined two color morphs and two source populations for the pollen donors. Bar heights represent the estimated marginal means from the GLMM and error bars represent the 95% confidence interval.

**Fig 7:**
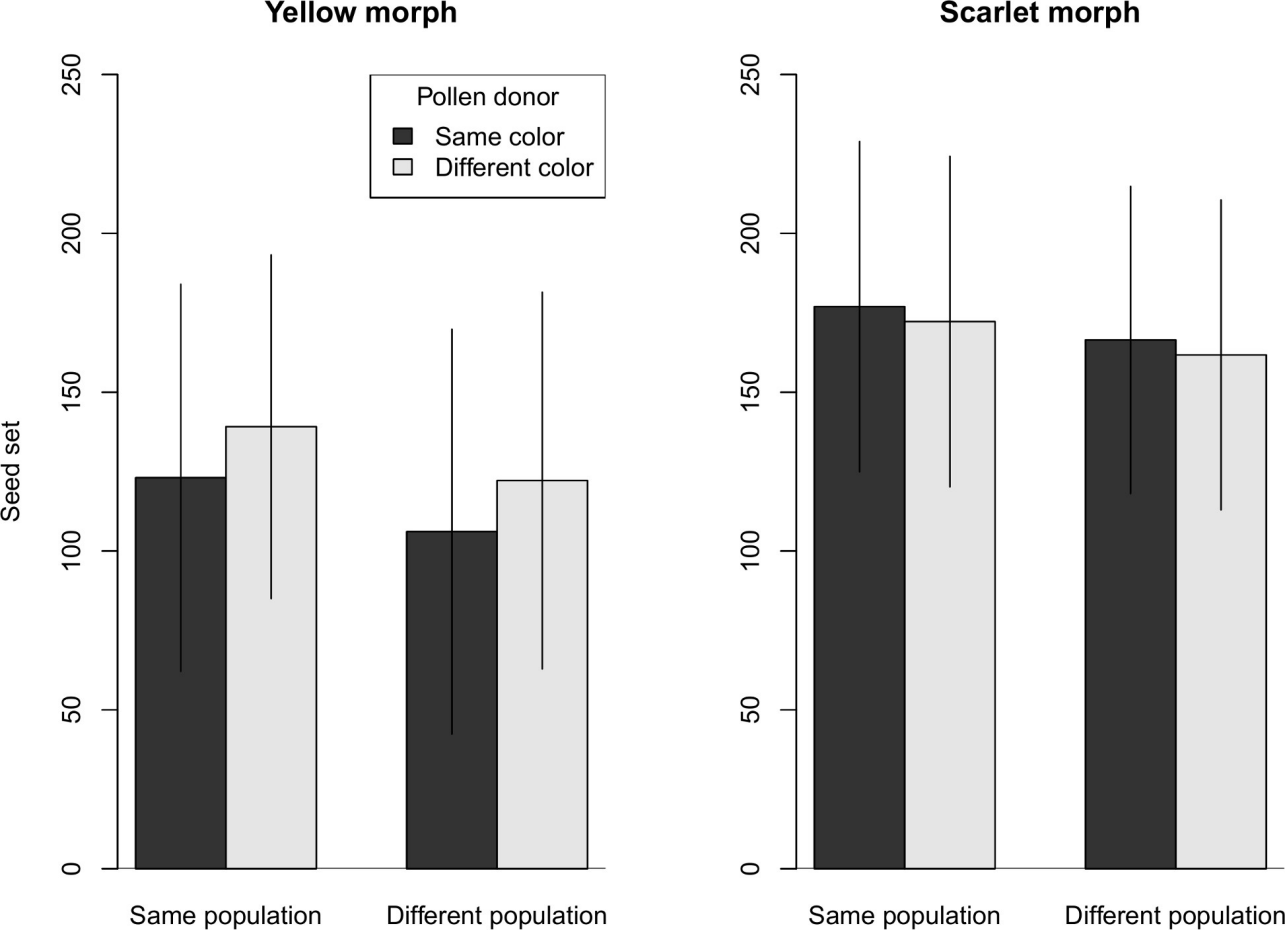
Seed set comparison in inter-population and inter-morph crosses. For each maternal color, we compared seed set in four treatments (SS, SD, DS, and DD) that combined two color morphs and two source populations for the pollen donors. Bar heights represent the estimated marginal means from the LMM and error bars represent the 95% confidence interval.

There was some evidence that fruit set was greatest for pollination between the same colors from same population (Fig 6, S1E Table). The manipulated movement of pollen between plants readily yielded fruits with a large number of seeds (Fig 7, S1F Table). In all combinations, pollination with same source population but different color saw a 6–15% reduction in fruit set. However, no reduction in seed set for pollinations using different population of different color was observed for maternal plants with yellow bracts, and the reduction was less than 3% for maternal plants with scarlet bracts (Fig 7). The “color” fixed effect did appear in the best candidate model for describing fruit set (Akaike weight = 0.417; Table 5), and the lack of interaction with maternal color suggests a consistent effect in both color morphs. The “color” fixed effect was not present in any of the top candidate models for seed set (best model that included “color”, ΔAICc = 2.16, Akaike weight = 0.113; Table 6).

**Table 5:** Summary of model selection results for fruit set and cross-compatibility between color morphs and populations.

Model	df	AICc	ΔAICc	Weight
Maternal color + Color + Pop + Maternal color: Pop	6	276.9	0	0.417
Maternal color + Pop + Maternal color: Pop	5	277.6	0.73	0.29
Maternal color + Color + Pop + Maternal color: Color + Maternal color: Pop	7	278.4	1.49	0.198
Pop	3	282.8	5.88	0.022
Null	2	283	6.12	0.02
Color + Pop	4	283.8	6.91	0.013
Color	3	284	7.07	0.012
Maternal color + Pop	4	284.8	7.93	0.008
Maternal color	3	285.1	8.17	0.007
Maternal color + Color + Pop	5	285.9	8.99	0.005
Maternal color + Color	4	286	9.12	0.004
Maternal color + Color + Maternal color: Color	5	287.4	10.47	0.002
Maternal color + Color + Pop + Maternal color: Color	6	287.5	10.62	0.002

Fruit set was predicted by three fixed effects and two of their interactions. The data for this set of models resulted from the SS, SD, DS, and DD treatments.

**Table 6:** Summary of model selection results for seed set and cross-compatibility between color morphs and populations.

Model	df	AICc	ΔAICc	Weight
Maternal color	4	1267	0	0.332
Null	3	1268	1.73	0.14
Maternal color + Same pop	5	1269	1.81	0.134
Maternal color + Same color	5	1269	2.16	0.113
Same pop	4	1271	3.74	0.051
Same color	4	1271	3.88	0.048
Maternal color + Same color + Same Pop	6	1271	4.02	0.045
Maternal color + Same pop + Maternal color: Same pop	6	1271	4.02	0.044
Maternal color + Same color + Maternal color: Same color	6	1271	4.17	0.041
Same color + Same pop	5	1273	5.93	0.017
Maternal color + Same color + Same Pop + Maternal color: Same color	7	1273	6.06	0.016
Maternal color + Same color + Same pop + Maternal color: Same pop	7	1273	6.27	0.014
Maternal color + Same color + Same pop + Maternal color: Same color + Maternal color: Same pop	8	1275	8.36	0.005

Seed set was predicted by three fixed effects and two of their interactions. The data for this set of models resulted from the SS, SD, DS, and DD treatments.

Fruit set was lower when pollen donors came from the scarlet population, whether the pollen donors were scarlet or yellow. The decline was 12–18% for yellow maternal plants (“different population” pollen donors) and 29–41% for scarlet maternal plants (“same population” pollen donors). The differential success of pollen from the two populations was reflected in the strong support for models that included the interaction between “population” and maternal color (top three combined Akaike weights = 0.905; Table 5).

Seed set was consistently 24–57% greater in maternal plants of the scarlet morph compared to the yellow morph (Fig 7), with seed set being slightly higher (by 6–16%) when the maternal plant and pollen donor came from the same population (Fig 7). Mixed-effects models provided little support for a role of pollen source in predicting seed set. Also, there is almost no support for an interaction between “population” and maternal color, as was found with fruit set. The variable with the strongest explanatory power for seed set was maternal color; the best performing candidate model had only maternal color as an explanatory variable (Akaike weight = 0.332, Table 6).

### Relative influence of fixed and random effects

For each statistical model, we estimated the amount of variance explained by the fixed effects (marginal *R*^2^) and the combination of fixed and random effects (conditional *R*^2^). The same random effect, a random intercept for individual plant, was present in all the models. When there is a large discrepancy between the marginal *R*^2^ and conditional *R*^2^, we expect a large influence of individual plant on the response variable. We found that the two *R*^2^ values were similar for all three of the fruit set models (Table 7), suggesting that the variance observed in fruit set was not explained by differences between individual plants. In the model testing the effect of pollinator exclusion on seed set (Table 4, Fig 5) the marginal *R*^2^ was more than half of the conditional *R*^2^ (Table 7); the fixed effects alone accounted for more than half of the variance observed in seed set. For the seed set models that tested self-compatibility (Table 2, Fig 3) and cross-compatibility (Table 6, Fig 7), the conditional *R*^2^ was much greater than the marginal *R*^2^ (Table 7), indicating a large influence of individual plants in the performance of the model.

**Table 7:** Comparison of the marginal (*R*^2^m) and conditional (*R*^2^c) coefficient of determination values.

	Response variable	Figure	*R*^2^m	*R*^2^c
Self-compatibility	Fruit set	Fig 2	0.0444	0.0444
	Seed set	Fig 3	0.151	0.441
Pollinator exclusion	Fruit set	Fig 4	0.136	0.145
	Seed set	Fig 5	0.286	0.464
Cross-compatibility and relative fitness	Fruit set	Fig 6	0.142	0.159
	Seed set	Fig 7	0.0758	0.456

*R*^2^m and *R*^2^c are marginal *R*^2^ and conditional *R*^2^, respectively.

## Discussion

*Castilleja coccinea* populations in the Midwestern region show intraspecific bract color polymorphism. Braum [49] reported that the color morphs were also associated with morphological differences, with the scarlet morph consistently larger in several bract and flower measurements including stamen and style length. Differences in both floral color and morphology could impact the breeding system of *C*. *coccinea* in ways that might involve reproductive trade-offs under pollen or pollinator limitation. Hence, we chose to investigate whether factors related to the breeding system might play a role in maintaining the floral color polymorphism. We found that both color morphs were self-compatible, and fruit set and seed set did not differ between selfed (SP) and outcrossed (SS) pollinations (Figs 2 and 3). Both color morphs are also inter-morph cross-compatible, although there may be evidence of a small reduction in fruit set in inter-morph crosses (Fig 6). Two notable differences were found between the color morphs. First, they differed in their response to pollinator exclusion. In the control treatments (bagged, no hand pollination), the scarlet morph showed reduced fruit and seed set, whereas the yellow morph did not (Figs 4 and 5). Second, the scarlet morph set more seed than the yellow morph (Fig 7).

The genus *Castilleja* includes both self-incompatible (*C*. *levisecta*, *C*. *linariaefolia*, *C*. *miniata*, *C*. *rhexiifolia*, *and C*. *sulphurea*) [61–63] and self-compatible (*C*. *attenuata*) [64] species, but breeding system had not been previously assessed in *C*. *coccinea*. Results of this study show that *C*. *coccinea* is highly self-compatible because fruit set and seed set were not reduced in individuals that received self-pollen compared to individuals that received outcross pollen (Figs 2 and 3). This pattern is true in both color morphs as shown by the self-compatibility indices which are above the threshold of 0.75 to be described as self-compatible, following Lloyd and Schoen [57]. While self-incompatibility assures the genetic and evolutionary benefits of outcrossing [65], self-compatible species have the advantage of reproductive assurance when pollen is limited [66], especially when inbreeding depression is low.

Comparison of the self-pollination treatment (SP) and the negative control (BN) showed that, surprisingly, the pollinator exclusion treatment had little effect on the yellow morph, which showed only slight or no reduction in either fruit set and seed set (Figs 4 and 5). The scarlet morph showed reductions in both measures of female fertility under the pollinator exclusion treatment. Thus, the yellow morph would likely experience an advantage of reproductive assurance in cases of pollinator limitation, and perhaps even pollinator absence. Fruit set from the negative control might have been the result of true autonomous self-pollination. In the greenhouse, *C*. *coccinea* did not set fruits or seeds (J. Fant, personal observation). This suggests that the autogamous selfing we observed might be due to accidental transfer of self-pollen to stigma (“bag-effect”). Alternatively, predatory insects such as aphids and thrips (squatters) that dwell in flowers may have caused “quasi-autonomous” pollination [57]. Whatever the mechanism of autogamy, the yellow morph outperformed the scarlet morph in the negative control treatment, indicating the yellow morph's ability to tolerate limited pollen delivery. While there are many cases where different color flower morphs attract different pollinators [8,27,63], to our knowledge this is the first report of color morphs differing in their dependence on pollinators for self-fertilization. The abundance of pollinators appears to act as a selective agent in *C*. *coccinea*, with the yellow morphs being favored when pollinator abundance is low.

There are different modes of self-pollination that offer different levels of reproductive assurance. Geitonogamy, where pollen is transferred between flowers, does not offer reproductive assurance because it relies upon the same pollinator activity as cross-pollination. Bagging experiments like ours investigate autogamous selfing (within flowers), but we did not investigate the precise timing and mechanism, factors that are important for determining the level of benefits provided by reproductive assurance [57].

Greater seed set was observed for the scarlet morph compared to the yellow morph, suggesting that the two morphs also differ in potential reproductive output. Regardless of the bract color of the pollen donor or which population the pollen came from, the scarlet individuals consistently produced more seeds per capsule (Fig 7). This difference was also observed for individuals that were self-pollinated (Fig 3) (except for the negative control as already noted).

Our study was not ideally designed to distinguish the effects of genotypic differences among individual plants from the effects of treatment, since we could not apply every treatment to every individual. The small number of individuals in each treatment compounds this limitation. However, we made efforts to statistically assess the relative influence of fixed effects (experimental treatments, maternal plant color) and random effects (individual plant) in the models using marginal and conditional *R*^2^ [59]. Where the difference between these values is large, there is the potential that genotypic differences between individual plants may be confounding our experimental findings. We found that differences in individual plants explained almost none of the variance observed in the fruit set analyses, as the marginal *R*^2^ and conditional *R*^2^ were nearly equal (Table 7). Additionally, for our analysis of pollinator exclusion and seed set, the marginal *R*^2^ was more than half the value of the conditional *R*^2^. These findings reinforce our conclusions regarding the differences in fruit set between bract colors and among experimental treatments. Also, the influence of pollinator exclusion on seed set (which differs between color morphs) is largely confirmed. However, the conditional *R*^2^ was much greater for the other two analyses of seed set. Conclusions regarding seed set and the self-compatibility experiment, or the cross-compatibility experiment, must be made with caution. We cannot rule out the possibility that genotypic differences between plants randomly assigned to treatment were the primary drivers of the patterns we observed for the latter two tests.

Our study was conducted over a single flowering season, so we cannot say whether the higher seed set for the red morph would be maintained over multiple years or varying conditions. Differences in seed set were observed between color morphs when five floral color polymorphic species (*Cirsium palustris*, *Digitalis purpurea*, *Holcus lanatus*, *Polygonum persicaria*, *and Vicia sepium*) were under drought and well-watered treatments [36]. Under a drought treatment, pink/purple morphs had greater seed set, but under a well-watered treatment, white morphs had greater seed set. The year of our study (2013) was extremely wet from April through June but had near average temperature and precipitation from June through August (National Temperature and Precipitations Maps obtained from NOAA).

We also observed differences in male fertility between populations, though not between color morphs. Plants from the scarlet population were poor pollen donors; hand-pollinations using pollen from the scarlet population individuals consistently resulted in lower fruit set (Fig 6). The lower fruit set was not related to bract color but rather related to the source population of the pollen donor because both color morphs were tested as pollen donors. The poor quality or low quantity pollen of the scarlet population likely reduces the overall male fertility of the scarlet population. We did not directly test pollen viability or pollen competition in this study, and we do not know the cause of the reduced pollen performance. Different pollen viability among color morphs has been observed in *Claytonia virginica* [67]. The reduced male fertility in the scarlet population might be due to higher inbreeding depression in that population [68], although we have no other evidence that suggests inbreeding levels or levels of genetic variability differ between the two populations.

While the color morphs were cross-compatible, there was slight evidence of reduced fruit set for inter-morph crosses (Fig 6). Reduced intermorph compatibility may lead to reproductive isolation and genetic divergence of the color morphs. For seed set, there was no evidence that inter-morph crosses produced fewer seeds per fruit (Fig 7). Further studies of pollinator behavior, mating system, and population genetics could reveal more about the reproductive interaction between the two morphs. We are currently conducting a genetic study, using a double digest Restriction-Site Associated DNA sequencing (ddRADseq) approach [69], to address gene flow between color morphs, and compare genetic structure and inbreeding across morphs and populations.

This study was limited to two populations that differed strikingly in bract color frequency. The populations were very close to each other, but undetected site-specific effects might exist. Further investigations at additional sites would be needed to confirm that the reproductive differences we observed between scarlet and yellow bract colored *C*. *coccinea* extend across the species range. Based on findings from these two sites, we posit that these reproductive trade-offs maintain the bract color polymorphism in *C*. *coccinea*, where pollinators are selective agents. The scarlet morph has greater potential reproductive output, but the yellow morph has greater reproductive assurance when pollinators are limited. In the absence of pollen limitation, both bract colors develop fruit equally well, but the scarlet morph would yield greater seed set. This appeared to be the situation for our open control, where scarlet plants produced many more seeds per fruit (though fruit set was similar between morphs). When pollinators are limited, the yellow morph may self-pollinate at a higher rate. The differences we observed between the color morphs might also have conservation implications, with the scarlet morph more susceptible to the negative consequences of pollinator declines due to pollution, habitat loss, or habitat degradation [70,71].

## Supporting information

S1 Table Sample sizes for fruit set and seed set analyses. We provide the sample sizes for our study as number of individuals, number of flowers for fruit set analyses, and number of fruits for seed set analyses.
(DOCX)
