# Supplementary material for: Reproductive trade-offs maintain bract color polymorphism in Scarlet Indian paintbrush (Castilleja coccinea)
Source: PLoS One. 2019 Jan 2;14(1):e0209176. doi: 10.1371/journal.pone.0209176 (PMC6314579; doi:10.1371/journal.pone.0209176)
Supplement: S1 Table — We provide the sample sizes for our study as number of individuals, number of flowers for fruit set analyses, and number of fruits for seed set analyses. (DOCX) [file pone.0209176.s001.docx]

**S1 Table. Sample sizes for fruit set and seed set analyses.** We provide the sample sizes for our study as number of individuals, number of flowers for fruit set analyses, and number of fruits for seed set analyses.

**Table A. Sample size for fruit set comparison between self-pollination and outcrossing.**

|  | Yellow population | | Scarlet population | |
| --- | --- | --- | --- | --- |
| Treatment | SP | SS | SP | SS |
| No. individuals | 3 | 2 | 3 | 3 |
| No. flowers | 22 | 11 | 43 | 32 |

**Table B. Sample size for seed set comparison between self-pollination and outcrossing.**

|  | Yellow population | | Scarlet population | |
| --- | --- | --- | --- | --- |
| Treatment | SP | SS | SP | SS |
| No. individuals | 3 | 2 | 3 | 3 |
| No. fruits | 16 | 9 | 14 | 10 |

**Table C. Sample size for fruit set comparison between bagged, no hand pollination and self-pollination.**

|  | Yellow population | | Scarlet population | |
| --- | --- | --- | --- | --- |
| Treatment | BN | SP | BN | SP |
| No. individuals | 3 | 3 | 3 | 3 |
| No. flowers | 31 | 22 | 33 | 43 |

**Table D. Sample size for seed set comparison between bagged, no hand pollination and self-pollination.**

|  | Yellow population | | Scarlet population | |
| --- | --- | --- | --- | --- |
| Treatment | BN | SP | BN | SP |
| No. individuals | 3 | 3 | 3 | 3 |
| No. fruits | 13 | 16 | 14 | 14 |

**Table E. Sample size for fruit set comparison in inter-population and inter-morph crosses.**

|  | Yellow population | | | | Scarlet population | | | |
| --- | --- | --- | --- | --- | --- | --- | --- | --- |
| Treatment | SS | DS | SD | DD | SS | DS | SD | DD |
| No. individuals | 2 | 3 | 3 | 3 | 3 | 3 | 3 | 3 |
| No. flowers | 11 | 40 | 29 | 35 | 32 | 34 | 31 | 39 |

**Table F. Sample size for seed set comparison in inter-population and inter-morph crosses.**

|  | Yellow population | | | | Scarlet population | | | |
| --- | --- | --- | --- | --- | --- | --- | --- | --- |
| Treatment | SS | DS | SD | DD | SS | DS | SD | DD |
| No. individuals | 2 | 3 | 2 | 2 | 3 | 3 | 3 | 3 |
| No. fruits | 9 | 12 | 6 | 13 | 10 | 10 | 26 | 26 |

**Table G. Sample size for fruit set comparison in the open control between the two populations.**

|  | OC Yellow | OC Scarlet |
| --- | --- | --- |
| No. individuals | 3 | 3 |
| No. flowers | 31 | 58 |

**Table H. Sample size for seed set comparison in the open control between the two populations.**

|  | OC Yellow | OC Scarlet |
| --- | --- | --- |
| No. individuals | 2 | 2 |
| No. fruits | 6 | 10 |
